# Reviewed article: Mota DM, Leitão LO, Silva RS, Fernandes CFR, Silva MT. Actions to mitigate ocular adverse events related to hair styling cosmetics in Brazil: a descriptive and correlational study (2022-2024). Epidemiol Serv Saude. 2025:34;e20240449

**DOI:** 10.1590/S2237-96222025v34e20240449.b

**Published:** 2025-05-02

**Authors:** Giulia Vaz da Silva

**Affiliations:** FOUSP, Saúde Coletiva

**Table:** 

Rating	Excellent	Good	Average	Below average	Poor
Interest		✓			
Quality	✓				
Originality		✓			
Overall	✓				

**Table:** 

Questionnaire	Yes	No	Not applicable
Does the manuscript contain new and significant information to justify publication?	✓		
Does the Abstract (Summary) clearly and accurately describe the content of the article?	✓		
Is the problem significant and concisely stated?	✓		
Are the methods described comprehensively?	✓		
Are the interpretations and conclusions justified by the results?	✓		
Is adequate reference made to other work in the field?	✓		

## Manuscript Structure:

Length of article is: Adequate

Number of tables is: Adequate

Number of figures is: Adequate

Please state any conflict(s) of interest that you have in relation to the review of this paper (state “none” if this is not applicable): None

## Comments

Apreciei a relevância do tema abordado e a originalidade da pesquisa. A metodologia utilizada é robusta, embora algumas partes poderiam ser mais detalhadas para facilitar a replicação dos resultados. Os resultados são significativos, mas sugiro uma discussão mais aprofundada sobre suas implicações no contexto atual da literatura.

Recomendo também uma revisão gramatical em algumas seções para melhorar a fluidez do texto. Em geral, o artigo apresenta um bom potencial para publicação após as revisões sugeridas. Agradeço pela oportunidade de revisar este trabalho. 

